# Post-activation performance enhancement effect of drop jump on long jump performance during competition

**DOI:** 10.1038/s41598-023-44075-w

**Published:** 2023-10-09

**Authors:** Devisson dos Santos Silva, Daniel Boullosa, Erika Vitoria Moura Pereira, Micael Deivison de Jesus Alves, Matheus Santos de Sousa Fernandes, Georgian Badicu, Fatma Hilal Yagin, Felipe J. Aidar, Leila Fernanda dos Santos, Hortencia Reis do Nascimento, Luca Paolo Ardigò, Raphael Fabricio de Souza

**Affiliations:** 1 Department of Physical Education, Federal University of Sergipe (UFS), São Cristóvão, Brazil; 2 Graduate Program in Physical Education, Federal University of Sergipe (UFS), São Cristóvão, Brazil; 3 Group of Studies and Research of Performance, Sport, Health and Paralympic Sports—GEPEPS, Federal University of Sergipe (UFS), São Cristóvão, Brazil; 4 Federal University of Mato Grosso do Sul, Campo Grande, Brazil; 5 College of Healthcare Sciences, James Cook University, Townsville, Australia; 6 Research and Development Department, iLOAD Solutions, Campo Grande, Brazil; 7 Graduate Program, Postgraduate Program in Neuropsychiatry and Behavioral Sciences, Federal University of Pernambuco (UFPE), Recife, Brazil; 8 Department of Physical Education and Special Motricity, Faculty of Physical Education and Mountain Sports, Transilvania University of Braşov, 500068 Braşov, Romania; 9 Department of Biostatistics and Medical Informatics, Faculty of Medicine, Inonu University, 44280 Malatya, Turkey; 10 Department of Teacher Education, NLA University College, Oslo, Norway

**Keywords:** Epidemiology, Biological techniques, Psychology

## Abstract

Drop jump is widely used in training sessions, aiming for chronic effects on long jump performance. However, the acute effect of drop jump on long jump performance through its use as a Conditioning Activity (CA) has not been explored. The objective of this study was to verify the Post-activation Performance Enhancement (PAPE) responses induced by successive Drop Jumps (DJ) on competitive long jump performance. Eleven male jumpers (19.0 ± 2.0 years; 178.0 ± 9.0 cm; 73.1 ± 8.9 kg; and personal record 5.78 ± 0.44 m) volunteered for participation. The athletes performed 5 drop jumps 2 min (1′45–2′15 min) before the second, and fourth attempt during official competition of state level, the attempts without the use of CA were considered controls. The performance of the second (5.63 ± 0.43 m), third (5.65 ± 0.46, g = 0.24) and fourth (5.71 ± 0.34 m) jumps performed after activation were higher than the first (5.54 ± 0.45 m) in the control condition, p = 0.02, and p = 0.01 respectively. Differences were also found in the take-off vertical velocity of the jump between the fourth (1.55 ± 0.21) and the first jump (1.30 ± 0.40), p = 0.006. Jump performance showed positive correlation with approach velocity, r = 0.731, vertical take-off velocity, r = 0.412, and take-off duration, r = 0.508. The mean performance in jumping post-activation (5.67 ± 0.38 m) was higher than that without the use of previous CA (5.59 ± 0.44 m), p = 0.02, g = 0.19. The use of DJs as a CA prior to the long jump promotes improvements in the performance of the jump, which can be explained by the increase in the take-off vertical velocity in the athletes.

## Introduction

Competitive performance is strongly affected by warm-up protocols, these have several goals to be achieved including Post-activation Performance Enhancement (PAPE)^1^. PAPE is characterized by improved performance in voluntary activities after the performance of a maximal, or near maximal, muscle contraction (i.e., a conditioning activity [CA])^2,3^. The use of these strategies has been shown to be effective in performance in sports or movements that involve explosive actions, such as sprinting, throwing, and jumping^3,4^. Although recent studies point to improved performance in endurance events^5^.

The long jump competition is based on an approach run with optimal velocity and a unilateral push. The distance of the jump is influenced by the horizontal running velocity plus the vertical velocity^6^. Success or failure in competition can be defined by centimeters. As an example, the difference in the jump of the bronze medalist and the eighth place in the 2022 World Athletics Championship (Eugene-USA) is only seven centimeters. Thus, the use of strategies is fundamental in improving sports performance in an acute way during warm-up. Therefore, CA in a competitive environment is accessible and should be prioritized^7^.

Plyometric exercises (PE) are commonly used as a strategy to promote PAPE^8,9^. The use of these exercises as CA does not require sophisticated equipment and is associated with the high recruitment of type II muscle fibers that have susceptibility to PAPE^7^. In this sense, Drop Jump (DJ) is one of the PE’s used as a strategy for performance improvement in Countemovement Jump (CMJ)^10^, sprint^11^, throws^12^, repeated sprints^13^ and recently also effective for cycling^8^. Few studies have investigated the use of the DJ in a competitive environment or in the performance of specific athletic tests^12,14,15^.

Some evidence indicated that the inclusion of a set of 5 DJs with the best RSI, at the end of the warm-up can improvement the 1000-m time in male runners^14^. Additionally, the use of 5 DJs with a drop height of 40 cm before a throwing action induces an increase in performance in individuals with a high percentage of type II muscle fiber area^12^. An improvement was also found in the performance of experienced throwers of various levels, one minute after performing three CMJs^15^. In this way, the use of PAPE in a competitive environment can be an effective strategy for optimizing competitive performance.

The use of DJ is effective in improving the rate of strength development, an important component of special strength for jumpers^16^. In view of this, DJ is widely used in training sessions, aiming for chronic effects on long jump performance^16^. However, the acute effect of DJ on long jump performance through its use as a CA has not been explored. This information is of great importance for coaches of jumpers, who seek strategies to improve the competitive performance of their athletes. Therefore, this study aimed to verify the PAPE responses induced by a set of 5 DJs with the best RSI on long jump performance.

## Materials and methods

### Study design

This investigation used the competitive environment through pre- and post-intervention assessments to verify the acute effect of consecutive DJs on competitive performance in the long jump event. The present study was performed on the outdoor athletics track for 2 weeks. Each participant was informed about the purpose, procedures, and risks of the study. All procedures were approved by the Research Ethics Committee of the University, Federal of Sergipe, and were performed according to the Helsinki declaration.

**Figure 1:**
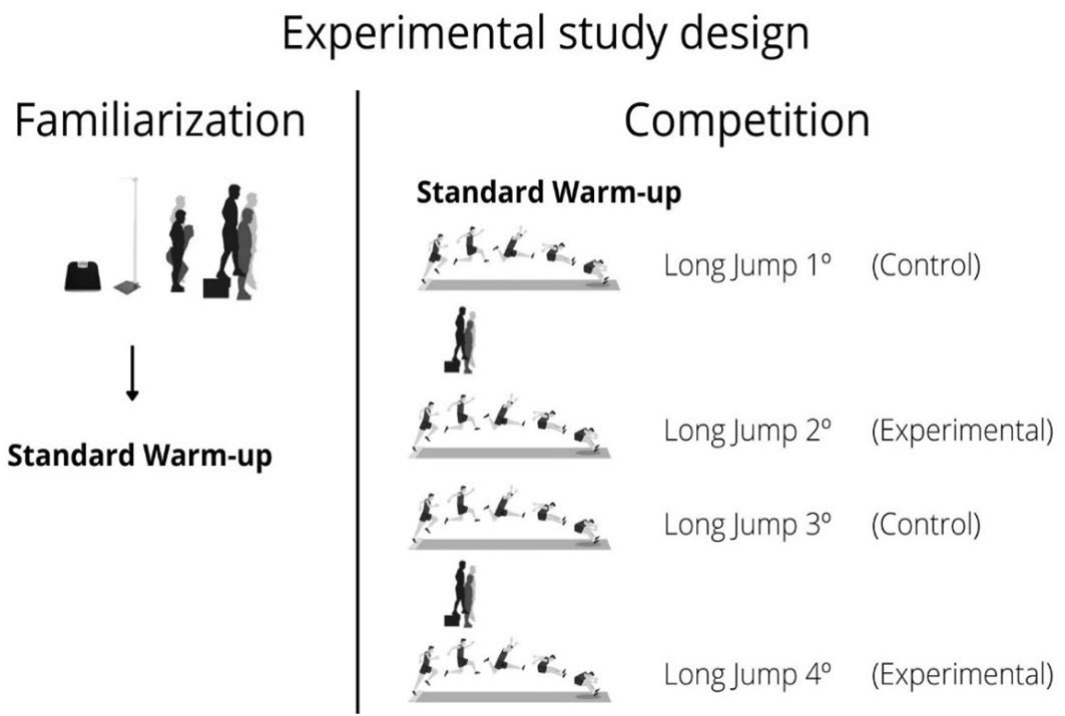
Experimental study design. *DJ* Drop Jump.

The subjects were evaluated at two different times: on the (i) first day, a familiarization session and anthropometric, CMJ and DJ assessment was performed; and on the (ii) second day, the individuals participated in a competition. Data collection was separated by 1 week, allowing for an appropriate recovery period (Fig. 1). In the competition, five DJs were performed as an CA strategy, two minutes before the second and fourth long jump attempts. The performances in these trials were compared with the results of the first and third trials in which no previous activation exercises were performed. Before these attempts, the athletes performed active recovery through running drills. Then, two minutes before the jumps, the athletes remained at rest and concentrated for the attempt. This experimental design is based on the study by Karampatsos et al.^15^, which showed positive results in track and field throwers. Approach velocity, take-off duration, vertical take-off velocity and horizontal take-off velocity were also evaluated.

### Subjects

The sample was composed of male long jumpers, participants in the Trota Mundo Long Jump Challenge, promoted by the Athletics Federation of Sergipe. Sample size was calculated a priori based on a statistical power (β = 0.80, assuming an effect size of 0.392 according to the findings of Karampatsos et al.^15^ and an alpha level of p < 0.05. The minimum sample size of eleven subjects was obtained (G × Power software package [version 3.1.9.4], Franz, Universitat Kiel, Germany). To be included in the study, subjects had to meet the following criteria (1) be aged 16 years or older, (2) perform at least three training sessions per week and have at least two years' training in track and field events (3) have at least six months experience in plyometric training, and (4) be free of musculoskeletal and joint injuries in the last six months. Thirteen male athletes were included in the study. All subjects were encouraged to continue their normal training except for the 24 h prior to the competition and the familiarization session. Alcohol and caffeine intake were not allowed before and during the experimental sessions. Exclusion criteria were any injury during the study period and absence from any session. All subjects signed an informed consent form. Parents of athletes under 18 years of age signed the informed consent form.

### Standard warm-up protocol

Athletes performed a warm-up during the familiarization session and on competition day. The protocol based on the usual warm-ups of the jumpers, included three minutes of light running on the track, dynamic stretching, running training, and long jump warm-up. Dynamic stretching was standardized, performing hip flexion/extension and hip adduction/abduction, with a series of 15 repetitions for each movement by body segment, with increasing execution velocity until the tenth repetition and maintaining the velocity until the end of the exercise. Running drills included 1 × 20-m (m) A-skips, B-skips, a-runs, in addition to performing 2 × 30 m of progressive running. The jumping drills consisted of 2 sets of 5 repetitions of take-offs every three strides, and 2 full approach runs with completion of the push-off, serving to gauge the running distance to the board. The warm-up lasted an average of 20 min.

### Long jump competition

The athletes were participating in an official state competition and performed four long jump attempts with a complete approach run, the interval between jumps was 15.6 ± 0.6 min. In the first 10 min of each 15-min interval, athletes performed a standardized active recovery that included dynamic stretches, 2 running progressions of 15 m, and technique-mimicking drills. This type of recovery is commonly used by long jumpers during competitions. In the control condition, after 10 min, the athletes rested and prepared for the next jump. For the experimental condition, the subjects performed the potentiation protocol whenever the second-to-last athlete ahead in the jump order started their attempt.

### DJ potentiation protocol

The athletes performed five DJs of the optimal drop height with a 15-s interval between jumps. The interval between the completion of the drop jumps and the long jump attempt was around 2 min (1′45–2′15 min). The use of this protocol was based on the studies by Chen^10^ and Zagatto^17^.

### Approach velocity and takeoff analysis

Approach velocity and takeoff analysis was evaluated using two pairs of photocells (Probotics Inc., USA) positioned at 11 and 1 m from the board. Contact time with the thrust board was calculated after filming using smartphone Iphone 8 (Apple Inc, Cupertino, CA, USA), being defined the number of frames of the time interval between the start and the loss of total contact of the foot with the ground, verified through the software Kinovea—version 0.9.5. All video records of the jumps were filmed (Canon EOS Rebel SL2) processed and analyzed using Kinovea—version 0.9.5. Anthropometric points (iliac crest and femoral condyle) were two-dimensionally fixed.

### CMJ and drop jump

#### CMJ evaluation

The CMJ evaluation was performed during the familiarization session. Performance in the CMJ was verified from the jump height (cm) that was estimated using the Chronojump-Boscosystem (Chronojump Bosco systems, Barcelona, Spain). The participant started in a standing upright position with his feet on a mat, approximately shoulder-width apart and with his hands on his hips. Then was performed a downward movement flexing his knees and hips to approximately 90°. Then he jumped vertically extending these joints. Everyone had three attempts, using 30 s rest between attempts. The highest jump was used for analysis.

#### DJ evaluation

During the familiarization session, subjects participated in a test to determine the optimal DJ drop height. Participants performed three DJs from two different heights (20 and 40 cm), the athletes were instructed to keep their hands on their hips and jump as high as possible with the minimum contact time. The optimal height was chosen using the reactive force index (jump height/contact time). The recording and analysis of the jump was done through the mobile application My Jump 2^18^ installed on Iphone 8 (Apple Inc., Cupertino, CA, EUA).

#### Long jump performance in state-level events

To examine if there is an effect of earlier attempts on later attempts in long jump performance during competitions held at the state level, we analyzed the results of the 2020, 2021, and 2022 Sergipan Athletics Championships (https://www.fsat.org.br/resultados). The analysis included only male participants who performed four valid jumps during each event. The final sample included 10 long jump athletes.

### Statistical analyses

Results were presented as mean ± standard deviation. The normality for each variable was verified using the Shapiro–Wilk test. The paired t-test was used to check for differences in mean long jump performance between the DJ and Control condition. Hedges (g) effect sizes (ESs) were calculated for each paired comparison and interpreted as trivial (< 0.20), small (0.20–0.49), moderate (0.50–0.79), large > 0.80^19^. A one-way ANOVA with repeated measures was used to identify if there was an influence of the time factor on the performance of the long jump and other variables. It was also used to verify if there was an influence of the time factor on the performance of the jump in the state competitions in the years 2020, 2021 and 2022. Mauchly's test of sphericity was applied, and the Greenhouse–Geisser. Epsilon correction was used when the sphericity criteria were not met. Size of main effects were calculated as partial eta squared (ηp^2^) and interpreted as small (0.01), medium (0.06), and large (0.14) effects^19^. The analyses were supplemented with Fisher's Least Significant Difference (LSD) post hoc test. Pearson's product moment correlation coefficient (r) was used to explore the relationships between the different variables. A significance level of p < 0.05 was set in all analyses. SPSS software version 25.0 (IBM Corp., Chicago, IL, USA) was used to carry out all the analyzes.

## Results

**Figure 2:**
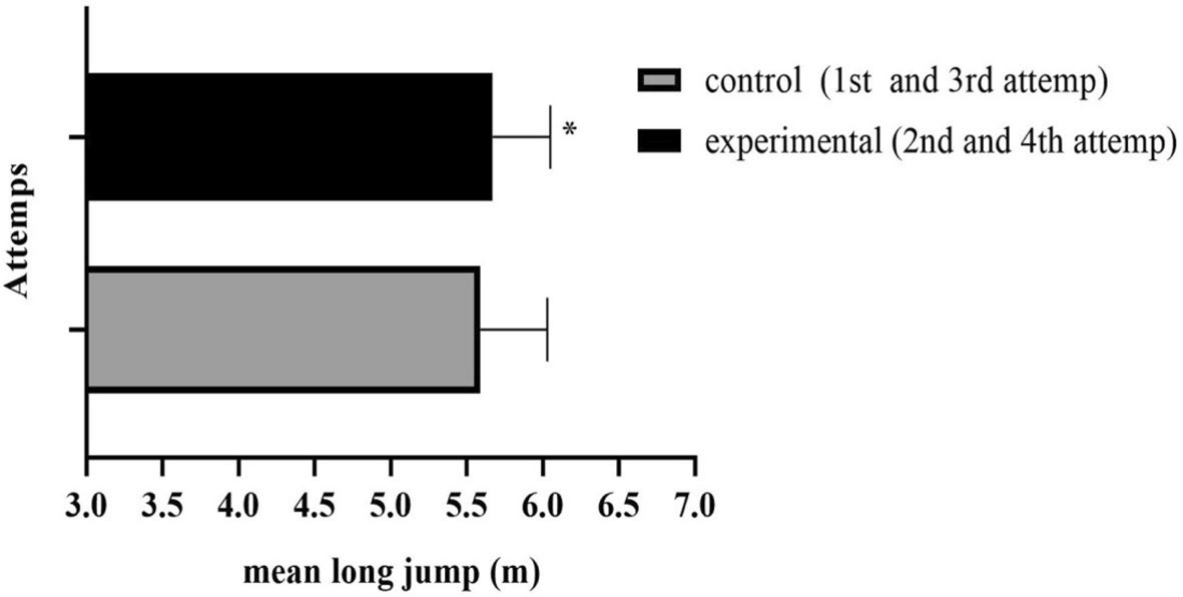
Comparison of average performance between control and experimental condition. *p < 0.05.

**Figure 3:**
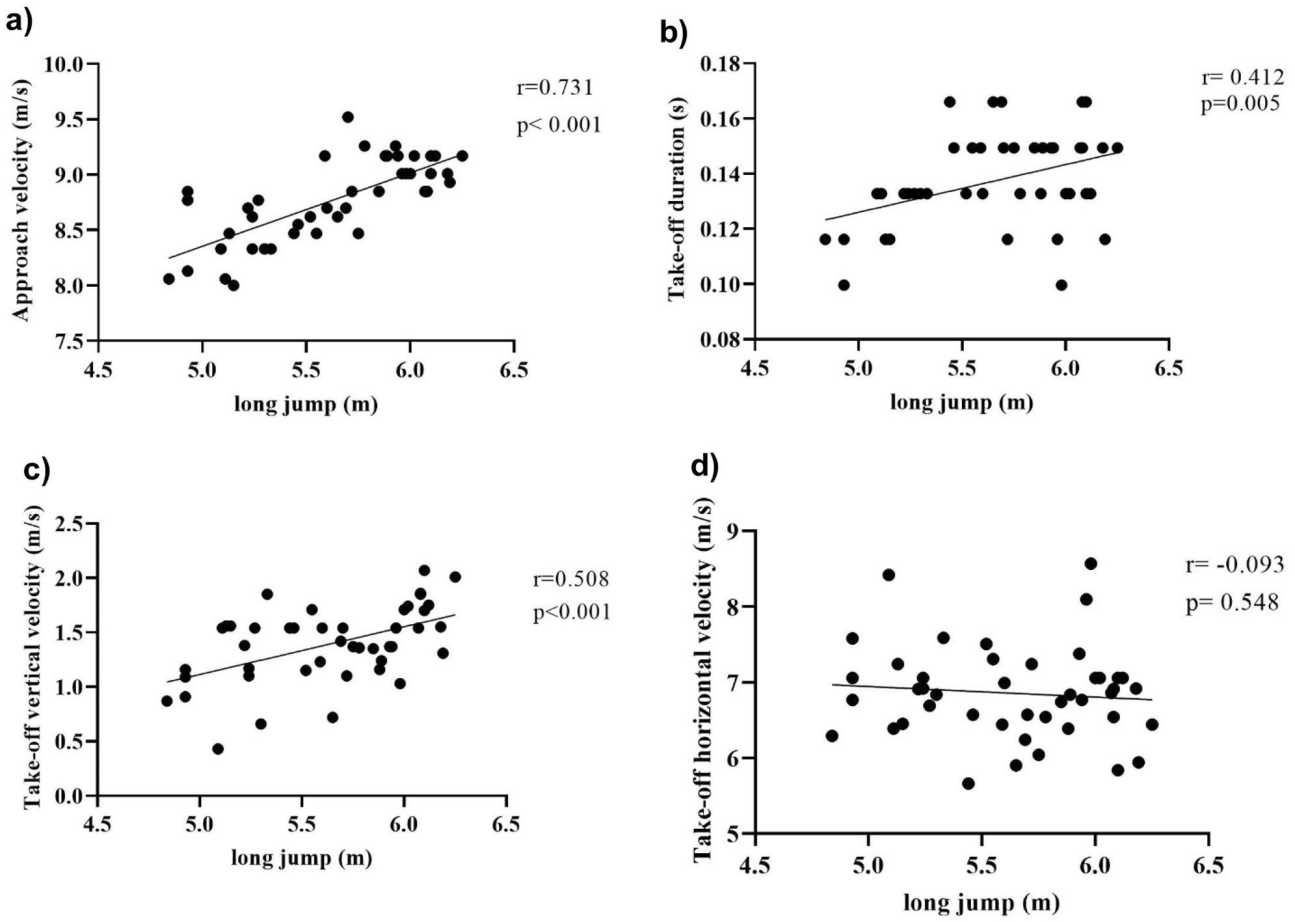
Long jump performance correlations: (**a**) Approach velocity, (**b**) Take-off duration, (**c**) Take-off vertical velocity, (**d**) Take-off horizontal velocity.

The final sample included 11 male jumpers. Two athletes who were excluded for not being able to make all jump attempts. Physical characteristics and training times are presented in Table 1. Through the paired t-test it was verified that the mean performance in jumping post-activation (5.67 ± 0.38) was higher than that without the use of previous CA (5.59 ± 0.44), p = 0.02, g = 0.19 (Fig. 2). ANOVA with repeated measures showed that there was a difference in jump performance between trials, through Fisher's LSD post hoc, it was observed that the second (5.63 ± 0.43, g = 0.20), third (5.65 ± 0.46, g = 0.24) and fourth (5.71 ± 0.34, g = 0.42) jumps performed after activation were greater than the first (5.54 ± 0.45) in the control condition, p = 0.02, and p = 0.01 respectively (Table 2). Differences were also found in the take-off vertical velocity between the four jump attempts. The post-hoc LSD found a difference in the take-off vertical velocity of the jump between the first and the fourth jump, p = 0.006. No significant differences were found in approach velocity, take-off horizontal velocity and take-off duration (Table 2). Jump performance showed positive correlation with approach velocity, vertical take-off velocity and take-off duration (Fig. 3). When analyzing the jump performance of the Sergipe athletes during the competitions in the years 2020, 2021, and 2022, no significant difference between attempts were observed [F (3,27) = 1.930; p = 0.14; ηp^2^ = 0.18].

**Table 1:** Physical and anthropometric characteristics.

Variables	Mean ± SD
Age (Y)	19.0 ± 2.0
Body mass (kg)	73.1 ± 8.9
Height (cm)	178.0 ± 9.0
CMJ height (cm)	38.8 ± 3.8
Training time (years)	3.5 ± 1.8
Personal record (m)	5.78 ± 0.44
RSI (cm/s)	2.6 ± 0.4

*cm* centimeters, *m* meters, *CMJ* Countermovement Jump, *kg* kilograms, *RSI* Reactive strength index, *s* seconds, *Y* Years.

**Table 2:** Mean (SD) performance in the long jump and variables analyzed.

	Condition				ANOVA		
	1st attempt (control)	2nd attempt (experimental)	3rd attempt (control)	4th attempt (experimental)	F (3,30)	P	η_p_^2^
Long jump (m)	5.54 (0.44)	5.63 (0.43) *	5.65 (0.46)*	5.71 (0.34) *	3.131	0.040	0.238
Approach velocity (m/s)	8.75 (0.37)	8.74 (0.38)	8.82 (0.42)	8.78 (0.36	0.687	0.567	0.064
Take-off duration (ms)	13.43 (1.89)	13.73 (2.11)	13.73 (1.30)	13.89 (1.70)	0.445	0.723	0.043
Horizontal velocity at take-off (m/s)	7.07 (0.62)	6.75 (0.84)	6.64 (0.47)	6.90 (0.62)	1.282	0.298	0.114
Vertical velocity at take-off (m/s)	1.29 (0.40)	1.40 (0.36)	1.38 (0.37)	1.55 (0.21) *	2.950	0.049	0.228

*p < 0,05, compared to 1st attempt by LSD post-hoc.

## Discussion

The objective of the present study was to verify the PAPE responses induced by successive DJs on the performance of long jump. Our results indicate that the use of DJs as a previous activity promotes improvement in the long jump performance when compared to the control condition. Through comparison between trials, it was found that the second and fourth trials using the potentiation protocol were higher compared to the first test, performed in the control condition. This suggests that the inclusion of successive DJs as an activity prior to long jump promotes acute performance improvement. To the best of my knowledge, this is the first study to use the DJ as a CA aiming PAPE in the long jump test.

The use of DJs proved to be effective as a CA for acute performance in the long jump competition in a competitive environment. Confirming the effectiveness of plyometric exercises as a stimulus prior to the main activity. This evidence is like the studies^20^, where decathletes had superior performance in the long jump when using three 2-legged rebound vertical jumps with maximal effort as a PAPE strategy, when compared to the control condition^20^. However, our sample confirmed the result through a specific jumpers and the study design comparing the jump performance between conditions at the same competitive moment, aiming for higher ecological validity. Moreover, the use of DJs allows evaluating the intensity of the jump performance through the drop height, whereas for vertical jumps with rebound the intensity was determined subjectively.

When analyzing the other variables, an increase in the vertical takeoff velocity was found when comparing the last attempt performed in the experimental with the first attempt in the control condition. A factor that may be a determinant of performance, because, at all acceleration speeds, the optimal takeoff strategy that produces the greatest jump distance is to generate near the maximum possible vertical velocity^21^. This finding also corroborates with Bogdanis et al.^20^, in which there was improvement in the performance of the jump when using the rebound vertical jump as CA and the vertical takeoff velocity was the variable that showed similar results. Nonetheless, the sample was composed of decathletes who were not experts in the long jump modality, our findings support this same indication of potentiating effect in expert athletes.

There was a positive correlation between approach velocity, vertical takeoff velocity and contact time with jump performance. Although a strong correlation between approach running and jumping distance is already known, the approach velocity and contact time did not significantly differ when comparing between conditions, regardless the differences in the performance of the jump. The non-influence of CA on approach speed and contact time can be explained by the technical aspects of long jump competition. As the athletes perform the attempt through an approach with optimal velocity and with the need to adjust the position of the body for the takeoff, this implies the execution of the approach run in an optimal margin of velocity, the increase in velocity would also imply changes in accuracy and positioning in the takeoff^6,22^. The contact time is related to the mechanical components of the jump, therefore, increasing or decreasing considerably the contact time would cause changes in the aspects related to elastic force and the stretching and shortening cycle at the moment of the jump^21^.

The improved performance in the jump can be explained by the improved rate of force development resulting from the PAPE effect, as athletes using similar contact time and approach velocity generated higher take-off vertical velocity and improved jump performance. The mechanisms supporting the PAPE are increased phosphorylation of the myosin light chain kinase^23,24^, changes in muscle temperature^23^ and the increased water content in skeletal muscle^23^, both factors have an influence on muscle contractility. Although there was an increase in the third attempt when compared to the first, regardless of the condition in which there was no stimulation, we believe that, in addition to a warming effect, the fourth attempt that was stimulated was vastly superior. This corroborates the hypothesis that PAPE has influenced performance.

Our results imply the possibility of using plyometric exercises with fall as a previous CA even in complex activities such as long jump that involves an approach run followed by a unilateral jump, being influenced by several factors to determine performance. Moreover, to indicating the use of these protocols in a competitive environment. Therefore, coaches should evaluate their athletes according to their experience in plyometric training and their competitive level to adapt the stimulus to each athlete. Researchers should explore national and international elite samples, to verify if stimuli through plyometric exercises can improve the performance. It is also possible to explore high jump and triple jump competitions, which involve different technical gestures than long jump competitions.

## Conclusion

The use of DJs as a CA prior to the long jump promotes improvements in the performance of the jump, which can be explained by the increase in the takeoff vertical velocity in the athletes. These results provide coaches with a strategy to be used in competitive environment, aiming at the acute increase of performance, without the use of additional equipment and complex configurations.

## Data Availability

The datasets generated during and analyzed during the current study are available from the corresponding author on reasonable request.
