# Author Correction: Post-activation performance enhancement effect of drop jump on long jump performance during competition

**DOI:** 10.1038/s41598-023-46708-6

**Published:** 2023-11-07

**Authors:** Devisson dos Santos Silva, Daniel Boullosa, Erika Vitoria Moura Pereira, Micael Deivison de Jesus Alves, Matheus Santos de Sousa Fernandes, Georgian Badicu, Fatma Hilal Yagin, Felipe J. Aidar, Leila Fernanda dos Santos, Hortencia Reis do Nascimento, Luca Paolo Ardigò, Raphael Fabricio de Souza

**Affiliations:** 1 Department of Physical Education, Federal University of Sergipe (UFS), São Cristóvão, Brazil; 2 Graduate Program in Physical Education, Federal University of Sergipe (UFS), São Cristóvão, Brazil; 3 Group of Studies and Research of Performance, Sport, Health and Paralympic Sports—GEPEPS, Federal University of Sergipe (UFS), São Cristóvão, Brazil; 4 Federal University of Mato Grosso do Sul, Campo Grande, Brazil; 5 College of Healthcare Sciences, James Cook University, Townsville, Australia; 6 Research and Development Department, iLOAD Solutions, Campo Grande, Brazil; 7 Graduate Program, Postgraduate Program in Neuropsychiatry and Behavioral Sciences, Federal University of Pernambuco (UFPE), Recife, Brazil; 8 Department of Physical Education and Special Motricity, Faculty of Physical Education and Mountain Sports, Transilvania University of Braşov, 500068 Braşov, Romania; 9 Department of Biostatistics and Medical Informatics, Faculty of Medicine, Inonu University, 44280 Malatya, Turkey; 10 Department of Teacher Education, NLA University College, Oslo, Norway

Correction to: *Scientific Reports* 10.1038/s41598-023-44075-w, published online 09 October 2023

In the original version of this Article Georgian Badicu was incorrectly affiliated with ‘Department of Physical Education, Federal University of Sergipe (UFS), São Cristóvão, Brazil’. Their correct affiliation is listed below.

Department of Physical Education and Special Motricity, Faculty of Physical Education and Mountain Sports, Transilvania University of Braşov, 500068, Braşov, Romania.

The original Article has been corrected.

